# The microbiota protects against respiratory infection via GM-CSF signaling

**DOI:** 10.1038/s41467-017-01803-x

**Published:** 2017-11-15

**Authors:** Rebecca L. Brown, Richard P. Sequeira, Thomas B. Clarke

**Affiliations:** MRC Centre for Molecular Bacteriology and Infection, Imperial College London, London, SW7 2AZ UK

## Abstract

The microbiota promotes resistance to respiratory infection, but the mechanistic basis for this is poorly defined. Here, we identify members of the microbiota that protect against respiratory infection by the major human pathogens *Streptococcus pneumoniae* and *Klebsiella pneumoniae*. We show that the microbiota enhances respiratory defenses via granulocyte–macrophage colony-stimulating factor (GM-CSF) signaling, which stimulates pathogen killing and clearance by alveolar macrophages through extracellular signal-regulated kinase signaling. Increased pulmonary GM-CSF production in response to infection is primed by the microbiota through interleukin-17A. By combining models of commensal colonization in antibiotic-treated and germ-free mice, using cultured commensals from the Actinobacteria, Bacteroidetes, Firmicutes, and Proteobacteria phyla, we found that potent Nod-like receptor-stimulating bacteria in the upper airway (*Staphylococcus aureus* and *Staphylococcus epidermidis*) and intestinal microbiota (*Lactobacillus reuteri*, *Enterococcus faecalis*, *Lactobacillus crispatus* and *Clostridium orbiscindens*) promote resistance to lung infection through Nod2 and GM-CSF. Our data reveal the identity, location, and properties of bacteria within the microbiota that regulate lung immunity, and delineate the host signaling axis they activate to protect against respiratory infection.

## Abstract (web summary)

The microbiota promotes resistance to respiratory infection through unclear mechanisms. Here, the authors identify members of the gut and upper airway microbiota that protect against respiratory infection by *Streptococcus pneumoniae* and *Klebsiella pneumoniae* by activating GM-CSF signaling.

## Introduction

The microbiota has an extensive influence on host physiology during homeostasis and disease. Changes in the bacterial composition of the mammalian microbiota, particularly in the intestine, have been linked to a variety of diseases and dysfunctions including autoimmunity, inflammatory disorders, and increased susceptibility to infection inside and outside of the intestine^1–4^. These conditions demonstrate the importance of indigenous bacterial communities for immune development and host defense.

For protection against intestinal infection, a variety of mechanisms have been described for how the microbiota inhibits the establishment of colonization by enteric pathogens and fortifies innate and adaptive defenses against those that are able to invade this site^5, 6^. In contrast, the mechanistic basis for how the microbiota protects against infection outside the intestine is limited. The microbiota has been shown to protect against systemic and respiratory infection by *Escherichia coli* ^7, 8^, influenza virus^9^, *Klebsiella pneumoniae* ^10, 11^, *Listeria monocytogenes* ^12^, *Staphylococcus aureus* ^13^, and *Streptococcus pneumoniae* ^14–16^. Despite a central role in promoting resistance to these pathogens, mechanistic understanding of the host cells, antimicrobial effectors, cytokines, and signal transduction pathways necessary to translate the effect of the microbiota into enhanced defenses to extra-intestinal infection, especially in the airway, are incompletely understood.

The facets of the microbiota important for the regulation of host defenses outside the intestine are also poorly defined. Studies using germ-free mice, and mice given multiple broad-spectrum antibiotics, have demonstrated that the microbiota as a whole protects against respiratory and systemic infection. Through the use of individual antibiotics and cohousing approaches to shift microbiota composition, a limited number of studies have proposed that certain members of the microbiota are associated with enhanced resistance to extra-intestinal infections and the regulation of extra-intestinal immunity^9, 17, 18^. Currently, however, the identity of the microbial groups in the microbiota that are directly responsible for enhancing respiratory defenses, why they have a dominant influence, and how they integrate with the required host signaling pathways to protect against extra-intestinal infection is unclear.

In this study, we sought to identify members of the microbiota that drive protection against respiratory infection, and to define the mechanistic basis for their effect. By combining microbiota depletion, gnotobiotic approaches with cultured commensal bacteria, and models of respiratory infection, we demonstrate that the microbiota promotes pulmonary clearance of Gram-positive and Gram-negative pathogens via granulocyte–macrophage colony-stimulating factor (GM-CSF). Intrapulmonary GM-CSF production in response to infection is regulated by the microbiota via interleukin-17A (IL-17A). Furthermore, we identify potent Nod-like receptor (NLR)-stimulating bacteria within both the upper airway and intestine that promote antibacterial defenses in the lung via this common mechanism.

## Results

### The microbiota protects against infection by diverse respiratory pathogens

**Fig. 1:**
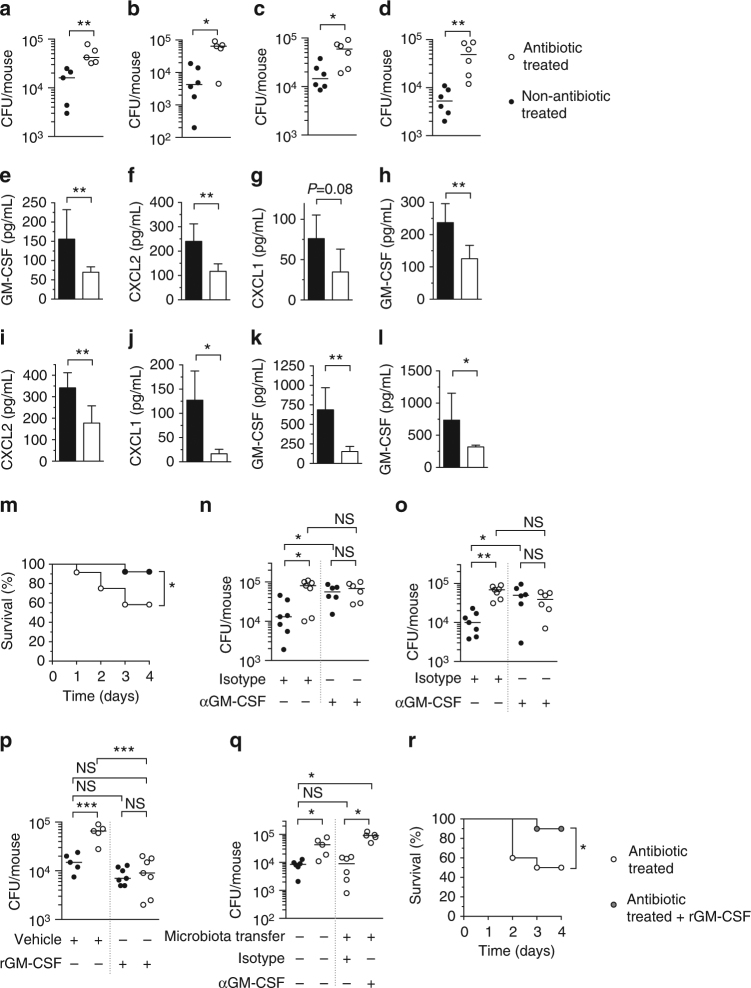
The microbiota protects against Gram-negative and Gram-positive lung infection via GM-CSF signaling. **a**, **b** *S*. *pneumoniae* and **c**, **d** *K*. *pneumoniae* burden 6 h (**a**, **c**) and 12 h (**b**, **d**) post-intranasal inoculation. **e**–**j** Cytokine levels in lung tissue 12 h post-inoculation with *S*. *pneumoniae* (**e**–**g**) and *K*. *pneumoniae* (**h**–**j**). **k**, **l** Cytokine levels in lung tissue 12 h post-intranasal inoculation with *K*. *pneumoniae* LPS (1 μg/mouse) (**k**) or lipoteichoic acid (LTA) (50 μg/mouse) (**l**). For (**e**–**l**) data are from *n* = 4–5 mice/group and error bars are s.d. Statistical comparisons were by Mann–Whitney (**a**–**f** and **h**–**l**) and Student’s *t*-test (**g**), *P < 0.05, **P < 0.01. **m** Survival after intranasal challenge with *S*. *pneumoniae* (*n* = 12–13 mice/group), groups were compared using the log-rank (Mantel–Cox) test, *P < 0.05. **n** *S*. *pneumoniae* and **o** *K*. *pneumoniae* burden in the lung 12 h post-intranasal inoculation. Indicated groups were intranasally administered a GM-CSF-neutralizing antibody, or isotype control (10 μg/mouse) (**n**,** o**), rGM-CSF, or vehicle control (5 μg/mouse) (**p**). **q** *S*. *pneumoniae* burden in the lung 12 h post-intranasal inoculation. Indicated groups were intranasally administered a GM-CSF- neutralizing antibody, or isotype control (10 μg/mouse) concomitant with bacterial challenge in the lung. For microbiota transfer, mice were intranasally inoculated with 10 μL of upper respiratory tract lavage fluid and orally inoculated with 200 μL of fecal suspension 72 h prior to *S*. *pneumoniae* infection. Each point represents a single mouse and horizontal lines indicate median values (**a**–**d** and **n**–**q**). Statistical comparisons were made by Student’s *t*-test with a post-hoc Sidak–Bonferroni correction for multiple comparison (**n**), and one-way ANOVA with post-hoc Sidak’s test (**o**–**q**) *P < 0.05, **P < 0.01, and NS, not significant. **r** Survival after intranasal challenge with *S*. *pneumoniae* ± rGM-CSF (5 μg/mouse daily) (*n* = 10 mice/group) groups were compared using the log-rank (Mantel–Cox) test, *P < 0.05

To begin to decipher the mechanism(s) by which the microbiota protects against respiratory infection, we compared the effect of the microbiota on the innate response to, and pulmonary clearance of, *S. pneumoniae* (Gram-positive) and *K. pneumoniae* (Gram-negative), both of which are major respiratory pathogens, but rely on different mechanisms to cause disease^19, 20^. To do this, mice were given antibiotics and then infected intranasally after antibiotic cessation. We confirmed that our antibiotic regimen caused a significant reduction in the bacterial load of the intestinal and upper airway microbiota (Supplementary Fig. 1a, b). In antibiotic-treated mice, there was an increase in the lung burdens of both *S*. *pneumoniae* (Fig. 1a, b), and *K*. *pneumoniae* (Fig. 1c, d) at 6 and 12 h post-inoculation, compared to non-antibiotic-treated controls. In line with these similar defects in bacterial clearance, there was reduced intrapulmonary production of GM-CSF, chemokine (C-X-C motif) ligand 2 (CXCL2), and CXCL1, critical signaling molecules in the innate response to respiratory infection, in antibiotic-treated mice infected with *S*. *pneumoniae* (Fig. 1e–g) or *K*. *pneumoniae* (Fig. 1h–j). Similarly, intrapulmonary cytokine production in response to innate stimulation of the lung with *K*. *pneumoniae* lipopolysaccharide (LPS), the major inflammatory component of this organism, was lower in antibiotic-treated animals, compared to non-treated controls (Fig. 1k). Innate production of intrapulmonary GM-CSF in response to lipoteichoic acid, a major inflammatory component of *S*. *pneumoniae*, was also reduced (Fig. 1l). Antibiotic-treated mice also suffered increased mortality during infection, confirming the importance of the microbiota for protection against respiratory infection (Fig. 1m). Collectively, these data demonstrate that the microbiota promotes a common innate response to respiratory infection by Gram-negative and Gram-positive pathogens, promotes their clearance from the lung, and enhances host survival during infection.

### The microbiota enhances respiratory immunity via GM-CSF signaling

Next, we wanted to understand how the microbiota drives this common innate response to respiratory infection and whether the effect of the microbiota on enhancing respiratory defenses was mediated by GM-CSF, CXCL1, or CXCL2. To test the role of GM-CSF, mice were administered a GM-CSF-neutralizing antibody concomitant with *S*. *pneumoniae* or *K*. *pneumoniae* infection. Antibody neutralization was used because GM-CSF-knockout mice have defects in alveolar macrophage development^21^. After GM-CSF neutralization, there was no difference in lung bacterial burden between antibiotic- and non-antibiotic-treated mice (Fig. 1n, o). Importantly, defects in pulmonary clearance were still evident in antibiotic-treated mice given an isotype control antibody (Figs. 1n, o). Bacterial clearance was restored in antibiotic-treated mice administered recombinant GM-CSF (rGM-CSF) concomitant with lung infection (Fig. 1p). Simultaneous transfer of the microbiota from the upper airway and gastrointestinal tract of non-antibiotic-treated into antibiotic-treated mice restored bacterial clearance in the lung (Fig. 1q). This effect of microbiota transfer was lost upon neutralization of GM-CSF, further demonstrating the role of GM-CSF in microbiota-mediated regulation of lung immunity (Fig. 1q). By contrast, defects in bacterial clearance from the lung persisted in antibiotic-treated mice after neutralization of CXCL1 (Supplementary Fig. 2a) and CXCL2 (Supplementary Fig. 2b). Effective neutralization of CXCL2 was confirmed by enzyme-linked immunosorbent assay (ELISA) (Supplementary Fig. 3). Antibiotic-treated mice given rGM-CSF showed significantly greater survival during bacterial lung infection, compared to antibiotic-treated mice given vehicle control (Fig. 1r). This suggests that GM-CSF is necessary to translate signals from the microbiota into enhanced bacterial clearance from the lung and is sufficient to restore antibacterial immunity after microbiota depletion.

### IL-17A primes GM-CSF during infection downstream of the microbiota

**Fig. 2:**
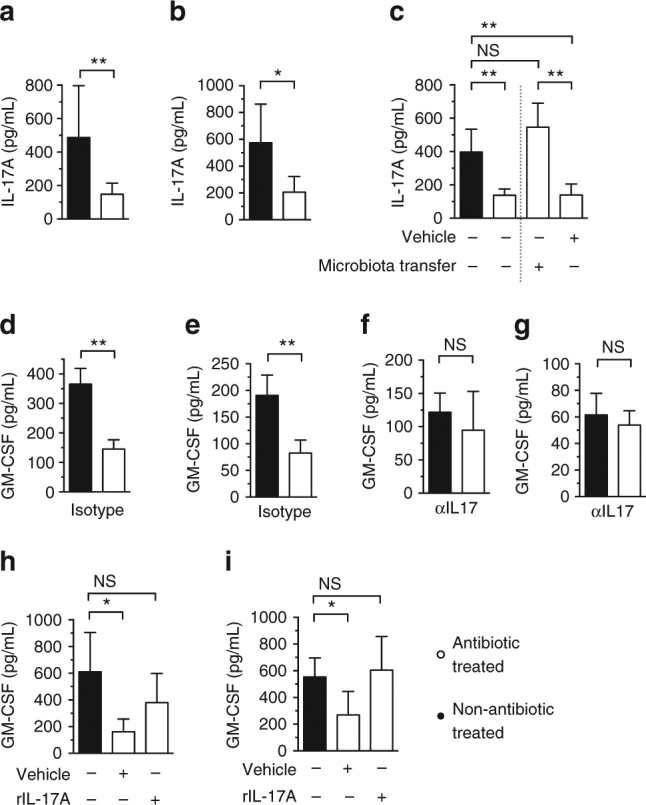
The microbiota primes GM-CSF production during infection through IL-17A. **a**–**c** IL-17A levels in lung tissue 12 h post-inoculation with *K*. *pneumoniae* (**a**) and *S*. *pneumoniae* (**b**,** c**). For microbiota transfer, mice were intranasally inoculated with 10 μL of upper respiratory tract lavage fluid and orally inoculated with 200 μL of fecal suspension 72 h prior to *S*. *pneumoniae* infection. **d**–**g** GM-CSF levels in lung tissue 12 h post-inoculation with *K*. *pneumoniae* (**d**, **f**) and *S*. *pneumoniae* (**e**, **g**). Indicated groups were intranasally administered an IL-17A-neutralizing antibody (100 μg/mouse via intraperitoneal injection), or isotype control 72 h prior to, and concomitant with, intranasal infection. **h**,** i** GM-CSF levels in lung tissue 12 h post-inoculation with *K*. *pneumoniae* (**h**) and *S*. *pneumoniae* (**i**). Indicated groups of mice were treated intranasally with rIL-17A, or vehicle control (5 μg/mouse) concomitant with bacterial challenge. Data (**a**–**i**) are from *n* = 5–6 mice/group and error bars are s.d. Statistical comparisons were by Mann–Whitney (**a**, **b**,** d**–**g**), or by one-way ANOVA with post-hoc Sidak’s or Dunnett’s test (**c**, **h** and **j**), *P < 0.05, **P < 0.01, ***P < 0.001, and NS, not significant

Having established the role of GM-CSF, we were then interested in determining how the microbiota regulates lung GM-CSF activity. In the intestine, IL-17A signaling is important for relaying signals from the microbiota to the immune system^22^. We therefore hypothesized that it could have a similar role in mediating the effect of the microbiota on GM-CSF signaling in the lung. To investigate this, first we analyzed lung IL-17A levels during respiratory infection. In antibiotic-treated mice there was significantly less intrapulmonary IL-17A produced in response to infection with *K*. *pneumoniae* (Fig. 2a) or *S*. *pneumoniae* (Fig. 2b), compared to non-antibiotic-treated mice. Simultaneous transfer of the microbiota from the upper airway and gastrointestinal tract of non-antibiotic-treated mice into antibiotic-treated mice rescued this defect in IL-17A production during lung infection (Fig. 2c), supporting the role of the microbiota in regulating lung IL-17A. To examine the role of IL-17A in regulating lung GM-CSF production during infection, mice were treated with an IL-17A-neutralizing antibody 3 days prior to, and concomitant with, lung infection. In mice treated with isotype control antibody there were still significant defects in GM-CSF production during lung infection of mice treated with antibiotics (Fig. 2d, e). After IL-17A neutralization, however, there was no longer any difference in GM-CSF production in response to lung infection between antibiotic-treated and non-antibiotic-treated mice (Fig. 2f, g). Furthermore, intranasal administration of recombinant IL-17A to antibiotic-treated mice rescued defects in respiratory GM-CSF production during infection with either *K*. *pneumoniae* or *S*. *pneumoniae* (Fig. 2h, i). Taken together, these data show that the microbiota promotes GM-CSF production during respiratory infection through IL-17A signaling.

### The microbiota regulates alveolar macrophage function via GM-CSF

**Fig. 3:**
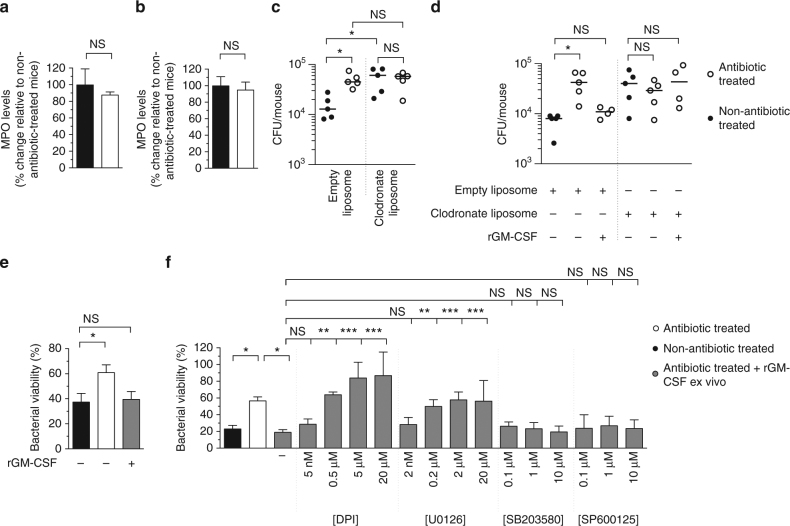
The microbiota promotes alveolar macrophage-mediated clearance of bacteria from the lung and the antibacterial activity of these cells is promoted by GM-CSF signaling via the ERK pathway. **a**,** b** Myeloperoxidase (MPO) levels in the lung 12 h post-inoculation with *S*. *pneumoniae* (**a**) and *K*. *pneumoniae* (**b**). Statistical comparisons were by Mann–Whitney, data are from *n* = 5 mice/group and error bars are s.d. (**a**, **b**). **c** *S*. *pneumoniae* and **d** *K*. *pneumoniae* burden in the lung 12 h post-intranasal inoculation. Indicated groups were administered liposome clodronate, or empty liposomes, 48 h prior to lung infection. rGM-CSF (5 μg/mouse) was given concomitant with intranasal inoculation with bacteria. Each data point represents a single mouse and horizontal lines indicate median value. **e**, **f** Alveolar macrophage killing of *S*. *pneumoniae* (**e**) or *K*. *pneumoniae* (**f**). Indicated groups of alveolar macrophages were treated with rGM-CSF (100 ng/mL) for 1 h and then either U0126 for 1 h, SB203580 for 1 h, SP600125 for 1 h, DPI for 30 min, or inhibitor vehicle control (DMSO, denoted by “−”) for 1 h prior to incubation with bacteria. Inhibitor concentration is displayed below each bar. Values are from *n* = 4 mice/group and error bars are s.e.m. Statistical comparisons were by one-way ANOVA with post-hoc Sidak’s or Dunnett’s test as appropriate (**c**, **e** and **f**) and Kruskal–Wallis test with Dunn’s multiple comparison test (**d**), *P < 0.05, **P < 0.01, ***P < 0.001, and NS, not significant

We next wanted to identify the innate cells programed by the microbiota via GM-CSF which effect this increase in pathogen clearance. Previous studies have found defects in lung-resident and recruited innate cells after microbiota depletion^10, 15^, but which innate effector cells are required for the microbiota-mediated enhancement of respiratory defenses is unknown. We found that neutrophil recruitment to the lung during infection by *S*. *pneumoniae* or *K*. *pneumoniae* (measured by myeloperoxidase (MPO) levels) is unaffected by antibiotic treatment (Fig. 3a, b). This is in accordance with our data showing that neutrophil-attracting chemokines (which were required to attract neutrophils to the lung during bacterial lung infection (Supplementary Fig. 4) did not mediate the effect of the microbiota on respiratory defenses (Supplementary Fig. 2a, b). This suggests that the microbiota-mediated enhancement of early respiratory defenses does not require neutrophils. This is supported by previous work suggesting that neutrophils are not required for the microbiota-dependent enhancement of *K*. *pneumoniae* clearance^10^. We therefore focused on the role of alveolar macrophages. To do this, we depleted alveolar macrophages using liposome clodronate. After antibiotic treatment, mice given empty liposome controls still had defects in the clearance of both *S*. *pneumoniae* and *K*. *pneumoniae* (Fig. 3c, d). In contrast, there was no difference in bacterial clearance between antibiotic-treated and non-antibiotic-treated mice after liposome clodronate treatment (Fig. 3c, d). This suggests that the effect of the microbiota on enhancing lung defenses to both pathogens requires alveolar macrophages. Crucially, rGM-CSF given to antibiotic-treated mice which were administered liposome clodronate no longer rescued defects in antibacterial immunity in the lung, whereas this rGM-CSF-mediated rescue was still evident in mice administered empty liposome controls (Fig. 3d). These observations indicate that the microbiota and GM-CSF work along a common pathway to promote alveolar macrophage-mediated clearance of bacterial pathogens from the lung.

### GM-CSF stimulates ERK to promote bacterial killing by alveolar macrophages

We then wanted to understand how the microbiota and GM-CSF promote pulmonary clearance of these pathogens by alveolar macrophages. Alveolar macrophage numbers were unaffected by antibiotic treatment (Supplementary Fig. 5), so we analyzed the bactericidal activity of these cells. In an ex vivo bacterial killing assay, alveolar macrophages from antibiotic-treated mice had significant defects in killing both *S*. *pneumoniae* (Fig. 3e) and *K*. *pneumoniae* (Fig. 3f), compared to the alveolar macrophages from non-antibiotic-treated mice. Pretreatment of these cells with rGM-CSF prior to incubation with bacteria rescued defects in bacterial killing (Fig. 3e, f), further supporting the role of GM-CSF in translating signals from the microbiota into enhanced host defenses. This led us to investigate how GM-CSF stimulation of alveolar macrophages rescued defects in bacterial killing after microbiota depletion. Downstream of GM-CSF receptor activation, mitogen-activated protein (MAP) kinases can play an important role in mediating changes in cell behavior^23^. To investigate the role of MAP kinases downstream of GM-CSF activation in our system, alveolar macrophages were treated with inhibitors of extracellular signal-regulated kinase (ERK) (U0126), c-Jun N-terminal kinase (JNK) (SP600125), and p38 MAP kinases (SB203580), over a range of concentrations, prior to incubation with rGM-CSF. Inhibition of the ERK, but not the JNK or p38 MAP kinase pathways, eliminated the GM-CSF-mediated rescue of bactericidal activity of alveolar macrophages from mice given antibiotics (Fig. 3f). We confirmed that GM-CSF activated ERK signaling in macrophages (Supplementary Fig. 6). To provide further support to the role of ERK in our model, we treated alveolar macrophages with a second ERK inhibitor, PD98059, prior to incubation with rGM-CSF. Again, this prevented GM-CSF-mediated rescue of bactericidal activity of alveolar macrophages from mice given antibiotics (Supplementary Fig. 7). Collectively, these data highlight a specific role for ERK signaling downstream of GM-CSF in our model. A critical antibacterial effector mechanism in innate immune cells regulated by MAP kinases is reactive oxygen species (ROS) production^24^. To examine the relationship between GM-CSF and ROS, alveolar macrophages from antibiotic-treated mice incubated with rGM-CSF were treated with the ROS inhibitor diphenyleneiodonium (DPI), over a range of concentrations. Compared to vehicle control, as DPI concentration increased this caused a significant reduction in bacterial killing by rGM-CSF-treated alveolar macrophages from antibiotic-treated mice (Fig. 3f). This correlated with reduced production of H_2_O_2_, as DPI concentration increased (Supplementary Fig. 8). Together, these data show that defects in the bactericidal activity of alveolar macrophages caused by microbiota depletion are rescued by GM-CSF, which requires downstream ERK signaling and ROS.

### PRR ligands from the microbiota enhance respiratory defenses via GM-CSF

**Fig. 4:**
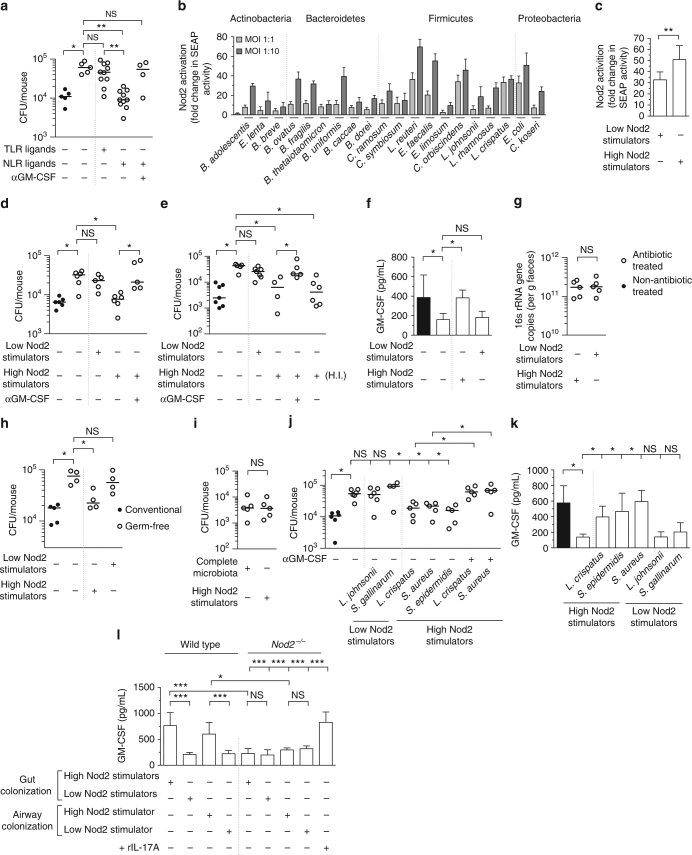
Potent NLR-stimulating bacteria in the upper airway and intestine regulate lung immunity through GM-CSF signaling. **a** *S*. *pneumoniae* lung burden 12 h post-intranasal inoculation. Indicated groups were orally inoculated with TLR ligands (LPS 50 μg and P3C 50 μg) or NLR ligands (MDP 50 μg and Mur*N*AcTriDAP 50 μ μg) 48 and 24 h prior to lung infection. Antibody treatment was as described in Fig. 1m. Statisitcal comparisons were by Kruskal–Wallis test with Dunn’s test (**a**). **b**,** c** NOD2-dependent SEAP production by HEK293 cells 30 h post-stimulation with individual members of the microbiota (**b**), or bacterial consortia at an MOI of 1:10 (**c**). Values are from 7 to 16 biological replicates and error bars are s.d. (**b**, **c**). **d**, **e** *S*. *pneumoniae* (**d**) and *K*. *pneumoniae* (**e**) lung burden 12 h post-intranasal inoculation. Mice were orally inoculated with indicated bacterial consortia (~5 × 10^8^ CFU) 48 and 24 h prior to intranasal infection. **f** GM-CSF levels in lung tissue 12 h post-inoculation with *K*. *pneumoniae*, data are from *n* = 5–8 mice/group and error bars are s.d. **g** 16 s rRNA gene copies in feces 3 days after oral inoculation with indicated consortia. **h** *S*. *pneumoniae* lung burden 12 h post-inoculation in outbred Swiss germ-free mice, and Swiss mice with a microbiota. Indicated groups were orally inoculated with bacterial consortia (10^8^ CFU) 72 h prior to *S*. *pneumoniae* infection. **i** *S*. *pneumoniae* lung burden 12 h post-intranasal inoculation. Indicated groups of mice received the complete microbiota as described in Fig. 1p or the “High Nod2-stimulating” consortia as described in Fig. 4d. **j** *S*. *pneumoniae* lung burden 12 h post-intranasal inoculation. Indicated groups of mice were intranasally administered 10^6^ CFU of indicated bacteria into the upper airway 48 h prior to inoculation with *S*. *pneumoniae* into the lung. Antibody treatment was as described in Fig. 1m. **k** GM-CSF levels in lung tissue 12 h post-inoculation with *S*. *pneumoniae*. **l** GM-CSF levels in lung tissue of wild-type and *Nod2* ^*−/−*^ mice 12 h post-intranasal inoculation with *K*. *pneumoniae* LPS (1 μg/mouse), data are from *n* = 5–8 and error bars are s.d. Indicated mice were orally inoculated with indicated bacterial consortia as described in Fig. 4d. For upper airway colonization, indicated mice were intranasally inoculated with 10^6^ CFU “High Nod2-stimulating” *S*. *aureus* and “Low Nod2-stimulating” *S*. *gallinarum*. Indicated groups were treated intranasally with rIL-17A, or vehicle control (5 μg/mouse) concomitant with *K*. *pneumoniae* LPS inoculation. Statistical comparisons were by Student’s *t*-test, with a post-hoc Sidak–Bonferroni correction for multiple comparison where appropriate (**d**–**k**), and one-way ANOVA with post-hoc Sidak’s test (**l**), *P< 0.05, **P < 0.01, ***P < 0.001, and NS, not significant. Each point represents a single mouse and horizontal lines indicate median values (**a**,** d**,** e**, **g**–**j**)

We then wanted to determine the molecular signals from the microbiota that enhance respiratory defenses through GM-CSF. Numerous studies have found that pattern recognition receptor (PRR) ligands from the microbiota regulate extra-intestinal immunity^2, 25^, but the host signaling pathways that convert these microbial signals into enhanced host defenses is unclear. Administration of peptidoglycan via the intestine that stimulates either of the peptidoglycan-recognizing NLRs, Nod1 or Nod2, restores pulmonary clearance of *K*. *pneumoniae* in antibiotic-treatmented mice^10^. We therefore wanted to know whether these NLR ligands also rescued defects in *S*. *pneumoniae* clearance caused by antibiotic treatment. Oral administration of NLR ligands restored *S*. *pneumoniae* clearance in antibiotic-treated mice to levels similar to non-antibiotic-treated controls, and NLR ligands were more effective than Toll-like receptor (TLR) ligands at mediating this rescue (Fig. 4a). Next, we wanted to determine whether GM-CSF mediated the effects of NLR ligands on lung defenses. In mice treated with antibiotics and given NLR ligands orally prior to lung infection, neutralization of GM-CSF abrogated the rescue of respiratory clearance (Fig. 4a). Similarly, we confirmed that NLR stimulation promoted *K*. *pneumoniae* clearance in a GM-CSF-dependent manner (Supplementary Fig. 9). These data, when considered with early data (Fig. 1n–q), demonstrate that the complete microbiota and NLR ligands from the gastrointestinal tract both regulate respiratory defenses via GM-CSF.

### Commensals that potently stimulate Nod2 regulate lung immunity via GM-CSF

This led us to try to identify bacterial species within the microbiota that enhance lung defenses and their site of colonization on the host. NLR-stimulating peptidoglycan is highly conserved in the bacterial phyla of the mammalian microbiota, so we thought that the ability to regulate lung immunity would not be restricted to a single bacterial species. Environmental and pathogenic bacteria, however, vary in their ability to stimulate NLRs^26^. This led us to hypothesize that members of the microbiota which are strong NLR activators would be significant regulators of antibacterial immunity in the lung. We chose to focus on Nod2 as a model NLR as this receptor recognizes peptidoglycan from both Gram-positives and Gram-negatives, compared to the more restricted Nod1 which recognizes peptidoglycan found predominately in Gram-negatives. To investigate our hypothesis, we cultured bacteria from four of the major phyla in the mammalian microbiota (the Bacteroidetes, Firmicutes, Actinobacteria, and Proteobacteria) and assessed their ability to stimulate Nod2. Using an in vitro HEK cell reporter expressing the Nod2 receptor, we found that these bacteria exhibited a large range in their ability to activate Nod2, from 2.6 ± 0.76-fold (*Eubacterium limosum*) to 36 ± 1.9-fold (*Lactobacillus reuteri*) above unstimulated controls (Fig. 4b). Furthermore, within each phylum there was a spectrum of Nod2 activation, suggesting, at least for the bacteria tested here, that there is no clear pattern of Nod2 activation that reflects taxonomic classification (Fig. 4b).

To begin to define members of the intestinal microbiota that regulate respiratory immunity we used this data to make consortia consisting of bacterial species common to the rodent and human intestinal microbiota^27–32^ that individually were weak stimulators of Nod2 at multiple multiplicities of infection (MOIs) (“Low Nod2-stimulating”: *Clostridium symbiosis*, *Eubacterium limosum*, *Lactobacillus johnsonii*, and *Lactobacillus rhamnosus*) and a second consortium that individually were strong Nod2 activators (“High Nod2-stimulating”: *Lactobacillus reuteri*, *Enterococcus faecalis*, *Lactobacillus crispatus*, and *Clostridium orbiscindens*). We tested the ability of these two groups to activate Nod2 and found that the “High Nod2-stimulating” consortium elicited significantly more Nod2-dependent nuclear factor-κB (NF-κB) activation than the “Low Nod2-stimulating” consortium (Fig. 4c). Additionally, we analyzed the activation of the major TLRs which recognize bacterial components by these consortia. We found that the “High Nod2-stimulating” and “Low Nod2-stimulating” consortia had similar TLR4 activating capacity and the “Low Nod2-stimulating” consortium had slightly, but not statistically significant, higher TLR2-stimulating capacity, compared to the “High Nod2-stimulating” consortium (Supplementary Fig. 10a, b). These data suggest that these consortia have similar capacity to stimulate bacterial sensing TLRs. Next, we tested the influence of these intestinal consortia on respiratory immunity in vivo. To do this, we orally inoculated either consortium into antibiotic-treated mice prior to infection with *S*. *pneumoniae* and *K*. *pneumoniae*. The “High Nod2-stimulating,” but not the “Low Nod2-stimulating,” consortium rescued defects in *K*. *pneumoniae* and *S*. *pneumoniae* clearance (Fig. 4d, e). Importantly, only the “High Nod2-stimulating,” and not the “Low Nod2-stimulating” consortium rescued defects in infection-induced GM-CSF production in antibiotic-treated mice (Fig. 4f). After oral inoculation of these consortia bacterial numbers in the intestine were equivalent, suggesting differences in lung immunity were not simply due to differences in bacterial load (Fig. 4g). Orally inoculated bacteria were not detected in the airways of mice (data not shown). The effect of the “High Nod2-stimulating” bacteria was lost upon GM-CSF neutralization (Fig. 4d, e). The “High Nod2-stimulating” group retained their ability to restore pulmonary immunity after heat inactivation (Fig. 4e), supporting the idea that the effects of these bacteria are due to their heat-resistant cell wall peptidoglycan and not their metabolic activity. To further demonstrate the importance of NLR-stimulating bacteria, germ-free mice were orally inoculated with either bacterial consortium prior to lung infection. Defects in bacterial clearance in germ-free mice were rescued by the “High Nod2-stimulating” but not the “Low Nod2-stimulating” consortium (Fig. 4h). These data confirm that the effects of the “High Nod2-stimulating” group were directly due to bacteria within this consortium and not to indirect effects of bacteria remaining in the intestine after antibiotic treatment. Furthermore, it suggests that this simple consortium provides equivalent protection against respiratory infection as a complete complex microbiota. In support of this latter proposition, we found that antibiotic-treated mice orally inoculated with the “High Nod2-stimulating” consortium had equivalent pathogen clearance from the lung as antibiotic-treated mice that had been administered with a complete microbiota from non-antibiotic-treated controls (Fig. 4i).

As our antibiotic regimen also depleted the upper airway microbiota (Supplementary Fig. 1b), next we wanted to investigate if bacteria colonizing this site regulate antibacterial immunity in the lung. If so, we were especially interested to determine if this microbial community proximal to the lung regulates pulmonary defenses via the same mechanism as the distal gastrointestinal microbiota. For this we intranasally inoculated mice with “High Nod2-stimulating” and “Low Nod2-stimulating” bacteria from the *Lactobacillus* and *Staphylococcus* genera (Fig. 4b), as bacteria from both of these taxa colonize the upper airway of humans and mice and can regulate immunity^33–37^. Inoculation of “High Nod2-stimulating” bacteria (*Lactobacillus crispatus*, *Staphylococcus aureus*, or *Staphylococcus epidermidis*) into the upper airway, but not “Low Nod2-stimulating” (*Lactobacillus johnsonii* or *Staphylococcus gallinarum*), rescued defects in pulmonary clearance of bacteria (Fig. 4j). The effect of the “High Nod2-stimulating” bacteria was abrogated by GM-CSF neutralization (Fig. 4j). Again, only the “High Nod2-stimulating,” and not the “Low Nod2-stimulating” consortium rescued defects in infection-induced GM-CSF production in antibiotic-treated mice (Fig. 4k). The ability of these upper airway bacteria to rescue lung immunity did not correlate with the ability to stimulate TLRs (Supplementary Fig. 10c, d). We confirmed that these commensals were present in the upper airway and that there was no difference in upper airway colonization levels, suggesting that the effects are not due to differences in bacterial load (Supplementary Fig. 11). We did not detect an increase in the levels of these intranasally inoculated commensals in the gastrointestinal tract, in comparison with non-intranasally inoculated controls (Supplementary Fig. 12a–c). As a further control to ensure that these intranasally inoculated airway commensals were signaling from the upper respiratory and not the gastrointestinal tract, we orally inoculated the “High Nod2-stimulating” upper airway bacteria into the gastrointestinal tract of antibiotic-treated mice. We used exactly the same commensal inoculation approach (dose and time before lung infection) that was used for upper airway colonization but we simply administered these commensals orally into the intestine and not the upper airway. In contrast to intranasal inoculation with the “High Nod2-stimulating” upper airway bacteria, oral inoculation of mice with these same bacteria did not rescue defects in antibacterial immunity after antibiotic treatment (Supplementary Fig. 13). Therefore, even if all bacteria that were inoculated into the upper airway went to the intestine, this would not be able to rescue defects in lung defenses. These data provide further support to the notion that “High Nod2-stimulating” upper airway commensals signal from the upper airway

Along those lines, next we wanted to confirm the importance of the Nod2 receptor in mediating the effect of these potent NLR-stimulating bacteria, whether colonizing the gastrointestinal tract or upper airway, on lung immunity. To do this, we gave wild-type and *Nod2* ^*−/−*^ mice antibiotics, then administered the “High Nod2-stimulating” and “Low Nod2-stimulating” gastrointestinal and upper airway commensals prior to respiratory challenge with *K*. *pneumoniae* LPS. In wild-type mice there was significantly higher GM-CSF production in response to *K*. *pneumoniae* LPS in mice administered the “High Nod2-stimulating” bacteria in the gastrointestinal tract, compared to those given “Low Nod2-stimulating” bacteria given via the same route (Fig. 4l). The same pattern was observed in antibiotic-treated wild-type mice administered a “High Nod2-stimulating” bacterium (*S*. *aureus*) in the upper airway, compared to upper airway administration of a “Low Nod2-stimulating” bacterium (*S*. *gallinarum*) (Fig. 4l). By contrast, there was significantly less GM-CSF produced in response to *K*. *pneumoniae* LPS in the *Nod2* ^*−/−*^ mice administered the “High Nod2-stimulating” bacteria via the gastrointestinal tract compared to wild-type treated with the same consortium (Fig. 4l). Similarly, there was significantly less GM-CSF produced in the *Nod2* ^*−/−*^ mice administered a “High Nod2-stimulating” bacterium via the upper airway compared to wild-type treated with the same organism. Furthermore, in contrast to antibiotic-treated wild-type mice, there was no difference in GM-CSF production between “High Nod2-stimulating” and “Low Nod2-stimulating” bacteria given via either route in antibiotic-treated *Nod2* ^*−/−*^ animals (Fig. 4l). These data confirm the importance of Nod2 in mediating the effect of these commensal bacteria on lung immunity. Critically, administration of recombinant IL-17A rescued defects in respiratory GM-CSF production in *Nod2* ^*−/−*^ mice, suggesting that *Nod2* ^*−/−*^ mice do not have any intrinsic defects in GM-CSF production, and further supporting the role of IL-17A in regulating GM-CSF production. Collectively, these data demonstrate that potent NLR-stimulating bacteria within the intestine and upper airway regulate lung immunity via a common GM-CSF-dependent pathway.

## Discussion

Our study fits within an emerging conceptual framework whereby the microbiota plays a significant role in determining the strength, duration, and type of immune response to infection^2, 38, 39^. In the lung, it has now been shown that resistance to multiple bacterial and viral pathogens is enhanced by the microbiota. This is often mediated by basal activity of PRRs and their cognate ligands from the microbiota^2, 25^. Here, we provide a number of additional mechanistic insights into the host factors and properties of the microbiota important for this. We elucidated a signaling axis involving IL-17A and GM-CSF by which the microbiota establishes a broadly protective innate defense program in the lung. In addition to coordinating host defenses, GM-CSF has a wider role in regulating homeostasis and allergic responses in the lung. The ability of the members of the microbiota identified in this study to modulate pulmonary GM-CSF signaling could therefore be of utility not only to promote resistance to infection but also to protect against allergy in the lung and interstitial lung disease in which GM-CSF can play a significant role. Alveolar macrophages are critical for this effect of the microbiota and it is their antibacterial capacity that is calibrated by the microbiota via GM-CSF that leads to enhanced pathogen clearance. We show that GM-CSF programs alveolar macrophage function via an ERK-specific signaling pathway leading to increased pathogen killing via ROS. Why this specific GM-CSF-ERK signal transduction pathway is used is currently unclear but could center on the assembly of the NADPH oxidase complex leading to increased ROS production upon pathogen encounter. IL-17A is a pivotal cytokine for translating the effects of the microbiota into increased resistance to intestinal bacterial infection^22^. Here, we expand the role for IL-17A by showing that it is required for microbiota-mediated stimulation of GM-CSF production during the innate response to pulmonary bacterial infection. In the intestine there are a number of cellular sources of IL-17A that are important for enhanced innate intestinal defenses, including T-helper type 17 cells. In the airway potential sources of this cytokine include γδ T cells, invariant natural killer T cells cells, and alveolar macrophages themselves. Currently, however, the source of this cytokine and the molecular mechanisms by which the microbiota regulates IL-17A production in our model remains to be determined.

We found that increased resistance to lung infection is promoted by bacteria in the upper airway and intestine that are strong activators of NLR pattern recognition receptors. Specifically, we identified potent Nod2-activating members of the microbiota that regulate antibacterial lung immunity. Given that either Nod1- or Nod2-stimulating peptidoglycan from the intestine regulates antibacterial immunity in the lung^10^, this redundancy means it is likely that potent Nod1-stimulating members of the microbiota could also be major regulators of lung immunity. Our data provide further support to the role of NLRs, and NLR-stimulating members of the microbiota, as central coordinators of immune homeostasis. In addition to its role in regulating antibacterial lung immunity described here, it has now been shown that the microbiota signals through NLRs to regulate neutrophil function in the bone marrow^14^, circulating neutrophil and inflammatory monocyte lifespan^17^, promote steady-state hematopoiesis^40^, support isolated lymphoid follicle development^41^, and promote adjuvant activity^37^. This suggests that potent NLR-stimulating commensals could be used to fine-tune immune function at both the mucosa and non-mucosal sites. The biochemical basis for the differences in NLR activation between different members of the microbiota is unclear but could be due to structural differences in the peptidoglycan between bacterial species leading certain groups to be particularly potent NLR activators or could be due to differences in the amount of peptidoglycan produced per bacterium. Previous studies have led to debate about which of the host’s resident microbial communities promote resistance to respiratory infection^15, 42^. We demonstrate that immune cells in the lung integrate signals from bacteria in the upper airway and intestine via a common mechanism to establish the host’s baseline resistance to respiratory infection. In our previous work, we found that inoculation of purified NLR ligands into the upper airway was insufficient to rescue defects in lung defenses after microbiota depletion. In our current work, we find that restoration of a population of colonizing bacteria in the upper airway, which potently activates NLRs, is sufficient to rescue defects in lung innate immunity after microbiota depletion. We believe this highlights the importance of stable microbial colonization, and the attendant continuous PRR activation this provides, for the regulation of lung immunity by the upper airway microbiota, in comparison to the brief, intermittent immune activation through ligand inoculation into the upper airway. There is precedent for the importance of whole bacteria, compared to purified ligands, in PRR-mediated effects on the immune system in the airway. It has been shown that supplementation of the airway with a preparation of whole bacteria signaling through TLR2 protects against respiratory influenza A virus infection, in comparison a single dose of a synthetic TLR2 ligand administered in the airway was less effective at mediating this protective effect against influenza^36^.

The flexibility of the respiratory immune system to integrate microbial signals from different mucosal sites suggests that in addition to upper airway and intestinal bacteria, any commensal organisms in the lung, whether as permanent residents or aspirated from the upper airway, could also have an impact on resistance to respiratory pathogens. Exploitation of these communities to protect against infection has been hampered by limited knowledge of which bacteria program host defenses and by what mechanism. In previous work, segmented filamentous bacteria present in the murine intestine have been associated with enhanced respiratory defenses^13, 18^. Whether these bacteria directly regulate lung defenses, or cause changes in microbiota composition that then lead to increased respiratory defenses is unclear. Additionally, given that these organisms are thought to be primarily murine restricted, their potential utility to combat respiratory infection in humans could be limited. By identifying a range of bacterial species that are common colonizers of the human upper airway and gastrointestinal tract which regulate lung immunity, our work expands the potential mucosal communities that could be manipulated therapeutically to regulate lung immunity and protect against infection. Importantly, our simple consortium of commensal bacteria provides equivalent protection against respiratory infection as a natural complex microbiota. This means that for therapeutic microbiota manipulation for the regulation of innate defenses in the lung at least, the complications of fecal microbiota transplantation could be avoided by using a simple bacterial consortium, or even single bacterial species, either in the upper airway or gastrointestinal tract. By demonstrating that a simple bacterial consortium is as effective as a complex microbiota at protecting against lung infection, it suggests that innate defenses in the lung may be less affected by changes in microbiota complexity than other elements of the immune system. For example, it has been demonstrated that increasing microbiota complexity correlates with increased myelopoiesis^43^ and, conversely, reduced circulating IgE levels^44^. However, more detailed studies using defined “High Nod2-stimulating” and “Low Nod2-stimulating” commensal communities of different complexities are required to fully address this question. Our work does imply that changes in the PRR stimulatory capacity of the microbiota, which can occur during microbiota development^45^, for example, could influence susceptibility to lung infection. This demonstrates the importance of understanding the functional effect of commensal microbes on host immunity and highlights the difficulty in correlating levels of different bacterial groups by sequencing alone with changes in host health, as the number of a given bacteria in the microbiota may not be a reflection of their influence on host immunity^46^. Collectively, by identifying bacteria in the microbiota that protect against respiratory infection and the mechanistic basis for their effect, our work paves the way for translational studies investigating the potential of these resident bacteria to combat respiratory infection which remains a major cause of human mortality.

## Methods

### Bacterial strains

Bacteria used in this study and their growth conditions are outlined in Supplementary Table 1.

### Mice

The use of mice was performed under the authority of the UK Home Office outlined in the Animals (Scientific Procedures) Act 1986 after ethical review by Imperial College London Animal Welfare and Ethical Review Body (PPL 70/7969). Wild-type C57BL/6 mice were purchased from Charles River (UK) and Swiss mice were purchased from Envigo (UK). Germ-free Swiss Webster mice were purchased from Taconic (Denmark). *Nod2* ^*−/−*^ (C57BL/6) mice were purchased from the Jackson Laboratories (USA). All mice were female and between 6 and 10 weeks old. Mice were normally housed no more than five per cage with Aspen chip 2 bedding with a 12 h light and 12 h dark cycle at 20–22 °C. Mice were randomly assigned to experimental groups. Water was provided *ad libitum* and mice were fed RM1 (Special Diet Services).

### Quantification of bacterial load in microbiota

To quantify bacterial loads in the upper airway, antibiotic-treated and non-antibiotic-treated mice were humanely killed, the trachea exposed, cannulated, and lavaged with 500 μL of sterile phosphate-buffered saline (PBS) which was subsequently collected from the nares. Lavage fluid was plated on tryptic soy agar supplemented with 5% sheep’s blood to quantify total bacteria, tryptic soy agar to quantify Staphylococcal species, and DeMan, Rogosa, and Sharpe agar to quantify Lactobacilli. After incubation overnight at 37 °C in aerobic conditions, colony-forming unit (CFU) counts were performed. To quantify bacterial loads in the gastrointestinal tract, stool from mice were collected immediately before antibiotic treatment started and on day 14 of antibiotic treatment. Stool was weighed and DNA extracted using the E.Z.N.A. Stool DNA kit (Omega Bio-Tek) as per the manufacturer’s instructions. Stool was homogenized by bead disruption using a FastPrep-24 instrument (MP Biomedicals) by 15 repeated pulses of 20 s vortexing and a subsequent 2 min recovery. All samples were then vortexed for 30 s to ensure complete disruption. Processing included the optional steps of incubating at 95 °C to ensure sufficient lysis of Gram-positive bacteria, and treatment with 100 μg of RNase A for 3 min at 37 °C to remove unwanted RNA. The DNA was then directly used for quantitative PCR or diluted one in ten for the more abundant phyla. The reaction mixture was 1 × SYBR Green PCR Master Mix (Thermo Fisher Scientific), 200 nM of each forward and reverse primer (Supplementary Table 2), and 2 μL template DNA in a total reaction volume of 10 μL loaded on a frosted 96-well plate (4titude). Quantitative PCR was performed on an Applied Biosystems StepOnePlus instrument and cycled 40 times with 15 s at 95 °C and 1 min at annealing temp (Supplementary Table 2). To determine approximate bacterial numbers, a standard curve was generated using plasmids containing a cloned 16s rRNA gene from a representative bacterial species (Supplementary Table 2). Plasmid copy numbers were calculated using the formula: (amount of plasmid in ng × 6.0221 × 10^23^ molecules/mol)/((4500 × 660 g/mol) × 1 × 10^9^ ng/g), where 4500 is the estimated length of the plasmid with the 16s rRNA gene insert and 660 g/mol is the average mass of 1 base of dsDNA. Using the standard curve, 16s rRNA gene copy number per gram of stool was determined.

### Models of lung infection and commensal inoculation

Mice were infected as described previously^10^. Briefly, mice were anesthetized and intranasally inoculated with approximately 10^5^ CFU of *K*. *pneumoniae* or *S*. *pneumoniae* in 50 μL of PBS. To determine bacterial load in the lung, mice were killed, lungs removed, homogenized in PBS, and plated on appropriate media. For survival curves, mice were killed when they exhibited two or more of the following signs of systemic pneumococcal infection: reduced movement, hunched posture, piloerection, shivering, dyspnea, or circling. No animal exhibited any of these signs of infection for more than 24 h. For innate stimulation of the lung, mice were anesthetized as above then intranasally administered with 1 μg of *K*. *pneumoniae* LPS (Sigma) or 50 μg lipoteichoic acid (Sigma). Alveolar macrophages were depleted using clodronate containing liposomes as described previously^47^. Antibodies and recombinant proteins were administered at amounts/concentration and times stated in figure legends. For microbiota transfer, unanesthetized mice were intranasally inoculated with 10 μL of upper respiratory tract lavage fluid and orally inoculated with 200 μL of fecal suspension 3 days prior to lung infection. Upper respiratory tract lavage fluid was prepared by lavaging the upper airway of non-antibiotic-treated mice, as described in “Quantification of bacterial load in gastrointestinal and upper airway microbiota,” the lavage fluid was centrifuged at 16,000 × *g* to pellet bacteria in the lavage fluid, the supernatant discarded and the pellet resuspended in 10 μL of sterile PBS. The fecal suspension was prepared by suspending a single fecal pellet from non-antibiotic-treated mice in 1 mL of sterile PBS. For oral inoculation of bacterial consortia, mice were orally gavaged at indicated time points with either consortia resuspended in sterile PBS. All bacteria were grown to mid-log phase. For intranasal inoculation of “High” and “Low” NLR-stimulating bacteria, unanesthetized mice were administered indicated bacteria in 20 μL, 48 h prior to lung infection. Because of the small inoculation volume and lack of anesthesia these bacteria remain in the upper airway^48, 49^.

### Microbiota depletion and antibody treatment

Mice were given antibiotics in drinking water as described previously^10, 15^. Mice were given broad-spectrum antibiotics (ampicillin 1 g/L, neomycin sulfate 1 g/L, metronidazole 1 g/L, and vancomycin 0.5 g/L) in drinking water for 10–14 days. Antibiotic therapy was stopped 3 days prior to infection. PRR ligands were prepared as described previously and administered by oral gavage^10^. Anti-GM-CSF (BioLegend), anti-CXCL2 (R&D Systems), anti-CXCL1 (R&D Systems), and isotype control, rGM-CSF (BioLegend) or rIL-17A (Thermo Fisher Scientific), were administered intranasally concomitant with *S*. *pneumoniae* or *K*. *pneumoniae* inoculation, unless stated otherwise. Anti-IL-17A (R&D Systems) was administered 3 days prior to infection and concomitant with infection via the intraperitoneal route.

### Alveolar macrophage bacterial killing assay

Alveolar macrophages were isolated and bacterial killing assays performed as described previously^10^. In indicated experiments, alveolar macrophages were treated with rGM-CSF (BioLegend), U0126, SB203580, SP600125 (Cell Signaling Technology), PD98059 (Sigma), or DPI (Sigma) prior to incubation with bacteria. Reactive oxygen species production was measured using the Amplex Red hydrogen peroxide assay kit (Molecular Probes) for H_2_O_2_.

### Cytokine and *F4/80* expression measurement in the lung

ELISA kits for GM-CSF, IL-17A (BioLegend), CXCL2, CXCL1, and MPO (all Thermo Fisher Scientific) were used according to the manufacturer’s instructions. To measure *F4/80* expression in the lung, cDNA was made from homogenized lung tissue using a high-capacity cDNA reverse transcription kit following the manufacturer’s instructions (Applied Biosystems). cDNA was used in qRT-PCR reactions using primers listed in Supplementary Table 1 with 1 × SYBR Green PCR Master Mix (Thermo Fisher Scientific). Expression of *F4/80* between groups was compared using the ΔΔC_T_ method relative to *Gapdh*. Primers used are given in Supplementary Table 2.

### HEK cell culture and stimulation

HEK Blue cells expressing human NOD2 (catalog number hkb-hnod2), TLR2 (catalog number hkb-htlr2), TLR4 (catalog number hkb-htlr4) (InvivoGen), and control HEK Blue Null cells (catalog number hkb-null) (InvivoGen) were cultured at 37 °C in 5% CO_2_ in Dulbecco’s modified Eagle's media supplemented with 10% (v/v) fetal bovine serum (Invitrogen), 100 units/mL penicillin, 100 mg/mL streptomycin, 30 mg/mL blastocidin, and 100 mg/mL zeocin. HEK Blue cells coexpress the indicated human pattern recognition receptor and NF-κB-inducible secreted embryonic alkaline phosphatase (SEAP) which is heat stable. In response to their cognate ligand these cells produce SEAP which can be quantified using QuantiBlue detection media (InvivoGen) at 620 nm. Control null cells express the NF-κB-inducible secreted embryonic alkaline phosphatase but do not express pattern recognition receptors. For cell stimulation, cells were seeded in a 96-well plate at approximately 2.5 × 10^4^ cells/well in 100 μL of growth media and left to adhere overnight. The cells were then stimulated with mid-log phase, heat-killed bacteria at a multiplicity of infection of 1:1 or 1:10 in 100 μl of fresh growth media, and incubated at 37 °C for 30 h. Fifty microliters of supernatant was then removed, incubated at 65 °C for 5 min to inactivate any endogenous alkaline phosphatase activity in the growth media, and added to 150 μL of QuantiBlue alkaline phosphatase detection media (InvivoGen) and absorbance at 620 nm measured after approximately 30 min. None of the bacteria tested in this study elicited any SEAP production from null cells, confirming that the SEAP produced by the cells expressing the indicated pattern recognition receptor is specific to its cognate ligand (data not shown).

### Western blotting

Cells were lysed in 50 mM Tris HCl (pH 7.4), 1 mM EDTA, 150 mM NaCl, 1% (v/v) NP40, 5 mM NaF, 0.25% deoxycholate, and 2 mM NaVO_3_, and samples were separated on a 12% polyacrylamide gel. Proteins were transferred to a PVDF membrane (Millipore) and probed with either anti-phospho-ERK1/2 (Cell Signaling) or anti-ERK1/2 (Cell Signaling) antibodies. All secondary antibodies were horse radish peroxidase conjugated, and membranes were developed with LumiGLO reagent (Cell Signaling). Uncropped western blot images are shown in Supplementary Fig. 14.

### Statistical analysis

All statistical tests were performed using the GraphPad Prism software. To compare differences between two groups, the Student’s *t*-test or the Mann–Whitney test were used as appropriate. For all other comparisons a one-way analysis of variance (with post-hoc Sidak’s test or Dunnett’s test), a Kruskal–Wallis test with Dunn’s multiple comparison test, or the Student’s *t*-test (with Sidak–Bonferroni post-hoc test for multiple comparisons) were used as appropriate. Survival between groups was compared using the log-rank (Mantel–Cox) test. *P*-values <0.05 were considered significant.

### Data availability

The data supporting the findings of the study are available in this article and its Supplementary Information files, or from the corresponding author upon request.

## Electronic supplementary material

Supplementary Information
