# Supplementary material for: The microbiota protects against respiratory infection via GM-CSF signaling
Source: Nat Commun. 2017 Nov 15;8:1512. doi: 10.1038/s41467-017-01803-x (PMC5688119; doi:10.1038/s41467-017-01803-x)
Supplement: Supplementary file 1 — Supplementary Information [file 41467_2017_1803_MOESM1_ESM.pdf]

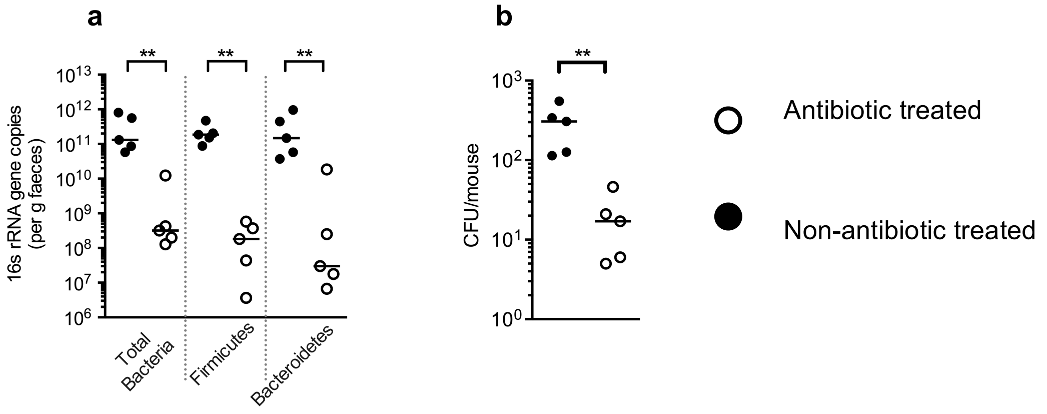

Supplementary Figure 1. a, 16s rRNA gene copies in mouse feces and **b**, number of viable bacteria in the mouse upper airway after 14 days of antibiotic treatment. Each point represents a single mouse and horizontal lines indicate median values, **$p < 0.01$ by Mann-Whitney.

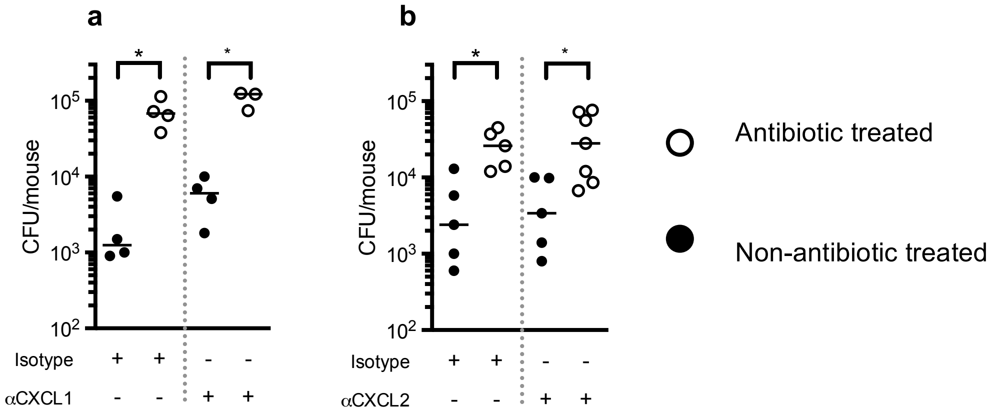

Supplementary Figure 2. *S. pneumoniae* burden in the lung 12 hours post-intranasal inoculation. Indicated groups of mice were intranasally administered a CXCL1 (a) or CXCL2 (b) neutralizing antibody, or isotype control (20 μg/mouse) concomitant with bacterial challenge. Statistical comparisons were by Kruskal-Wallis test with Dunn's multiple comparison test, *$p < 0.05$.

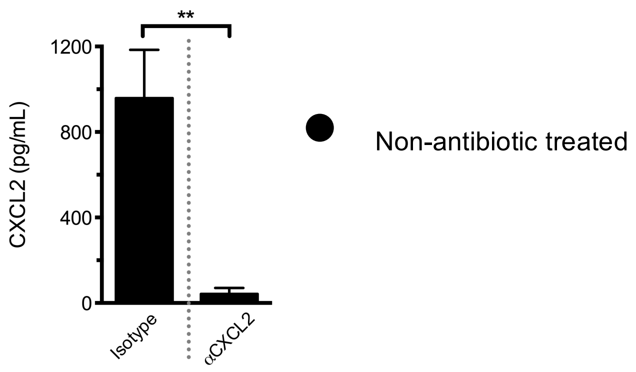

Supplementary Figure 3. CXCL2 level in the lung 12 hours post-intranasal inoculation with *S. pneumoniae* ±CXCL2 neutralizing antibody (20 μg/mouse) concomitant with infection. Data is from n=4-5 mice/group and error bars are s.d., **$p < 0.01$ by Mann-Whitney.

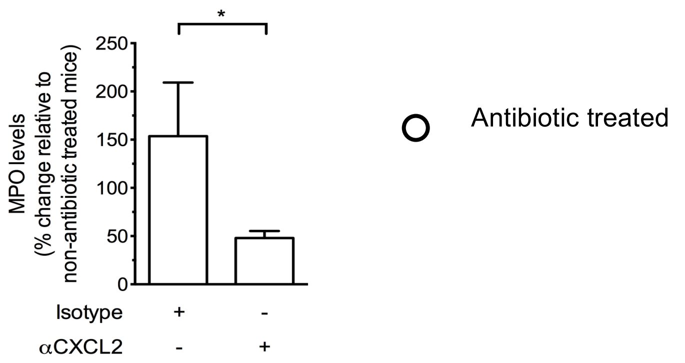

Supplementary Figure 4. Change in neutrophils levels (as measured by MPO) during *S. pneumoniae* infection in the lung of antibiotic treated mice after chemokine neutralization. Indicated groups of mice were treated with antibodies as described in **Figure 1** of our manuscript. Data is from n=5 mice/group, error bars are s.d. and $*p < 0.05$ by Mann-Whitney.

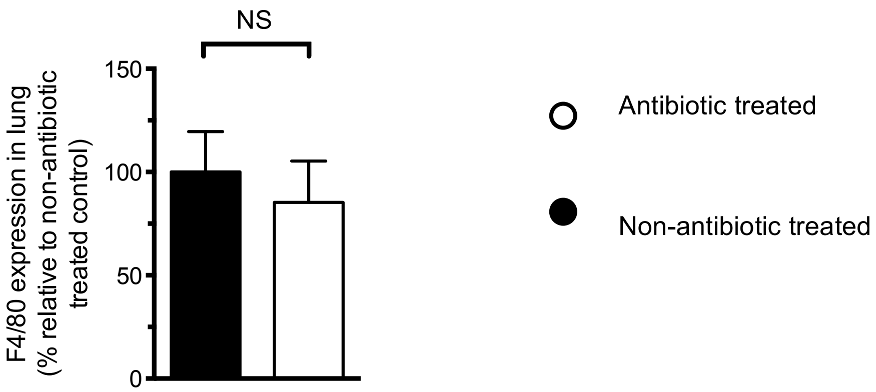

Supplementary Figure 5. The microbiota does not influence macrophage levels in the lung. Expression of the macrophage marker *F4/80* in lung tissue of wild-type mice either given antibiotics or non-antibiotic treated controls. Data is from n=4 mice/group, error bars are s.d. and NS is not significant by Mann-Whitney.

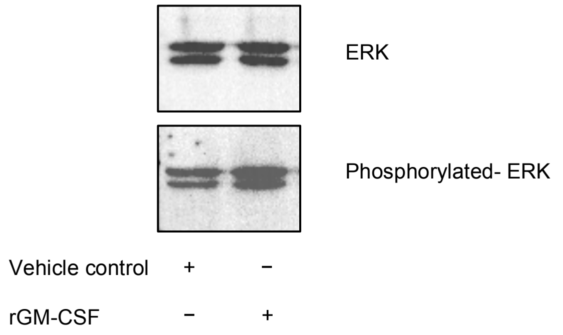

Supplementary Figure 6. GM-CSF stimulation induces ERK phosphorylation in macrophages. Western blot showing that recombinant GM-CSF (100 ng/mL, for 5 minutes) activates ERK signalling, as measured by ERK phosphorylation, in macrophages. Uncropped western blots are shown in **Supplementary Fig. 14**.

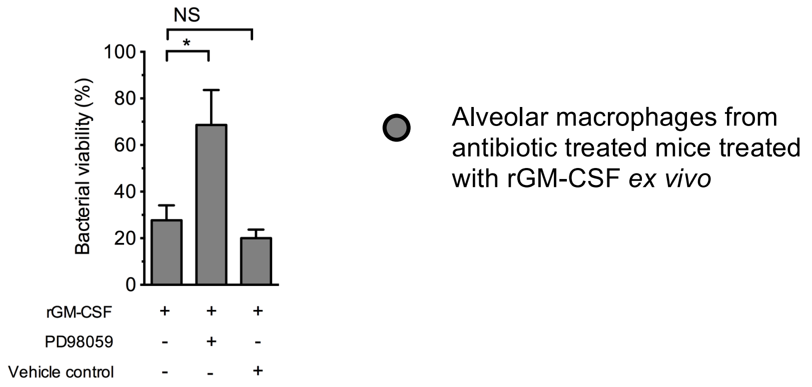

Supplementary Figure 7. The ERK inhibitor, PD98059, inhibits the stimulatory effect of GM-CSF on alveolar macrophage killing of bacteria. Alveolar macrophage killing of *K. pneumoniae*, as described in **Figure 3**. Indicated groups of alveolar macrophages were treated with PD98059 (5 μM), or vehicle control for 30 minutes, prior to rGM-CSF (100 ng/mL) for 1 hour, then *K. pneumoniae* were added. Data from n=4 mice/group, error bars are s.e.m. and $^*p < 0.05$ NS, not significant by one way ANOVA with post hoc Dunnett's test.

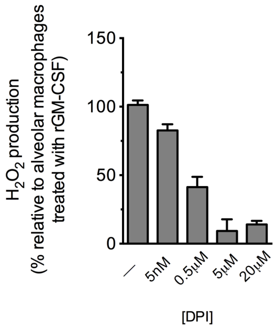

Supplementary Figure 8. ROS production by alveolar macrophages from antibiotic treated mice stimulated with rGM-CSF and incubated with *K. pneumoniae*. Macrophages where treated with indicated inhibitors as outlined in **Figure 3**, prior to treatment with rGM-CSF. H_2O_2 was measured as a proxy for ROS. Data from n=3 mice/group, error bars are s.e.m.

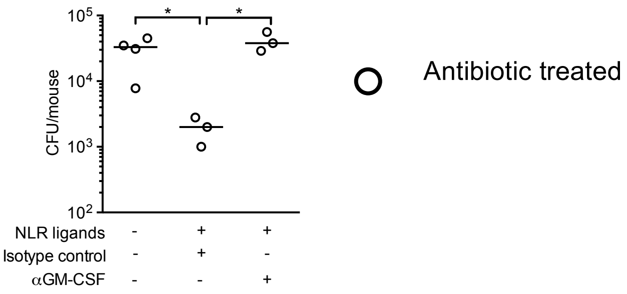

Supplementary Figure 9. *K. pneumoniae* burden in the lung 12 hours post intranasal inoculation. Indicated groups of mice were orally inoculated NLR ligands (MDP 50 µg and MurNAcTriDAP 50 µg) 48 and 24 hours prior to lung infection. Antibody treatment was as described in **Figure 1m**. Each point represents a single mouse, horizontal lines indicate median values, and statistical comparisons were by one way ANOVA with post hoc Sidak's test, *p < 0.05.

Intestinal colonizers

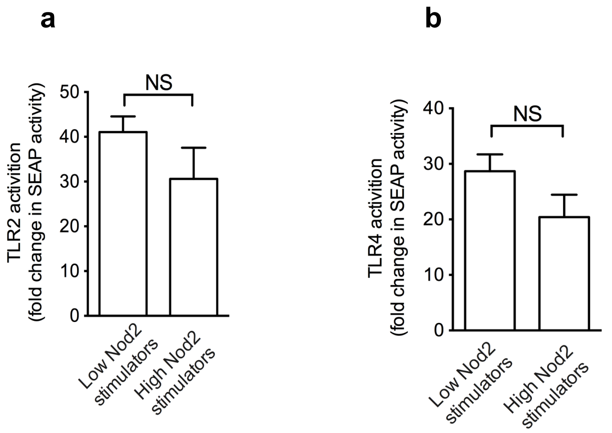

Upper airway colonizers

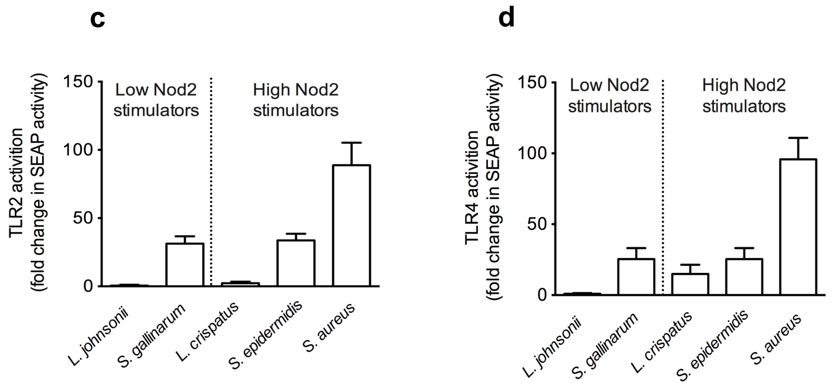

Supplementary Figure 10. a-d, TLR-dependent SEAP production by HEK293 cells 24 hours post-stimulation with indicated bacterial consortia at an MOI of 1:10. Values represent 5-16 independent biological replicates, error bars are s.d., NS not significant by Mann-Whitney.

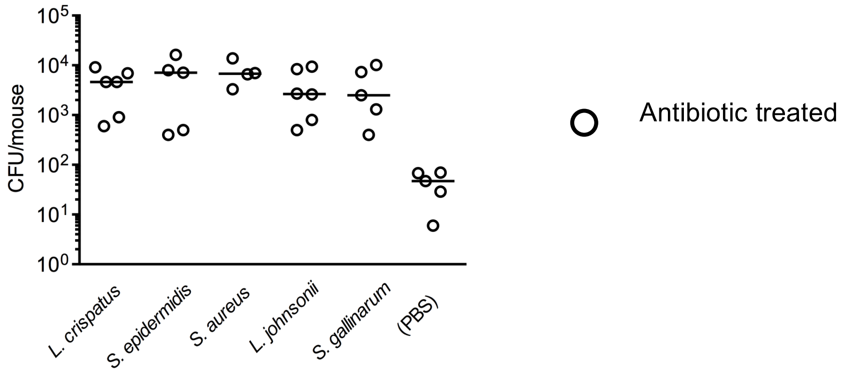

Supplementary Figure 11. Number of viable commensal bacteria in the upper airway of antibiotic treated mice 48 hours post-intranasal inoculation with indicated commensals (10^6 CFU), or vehicle control. Each point represents a single mouse and horizontal lines indicate median values.

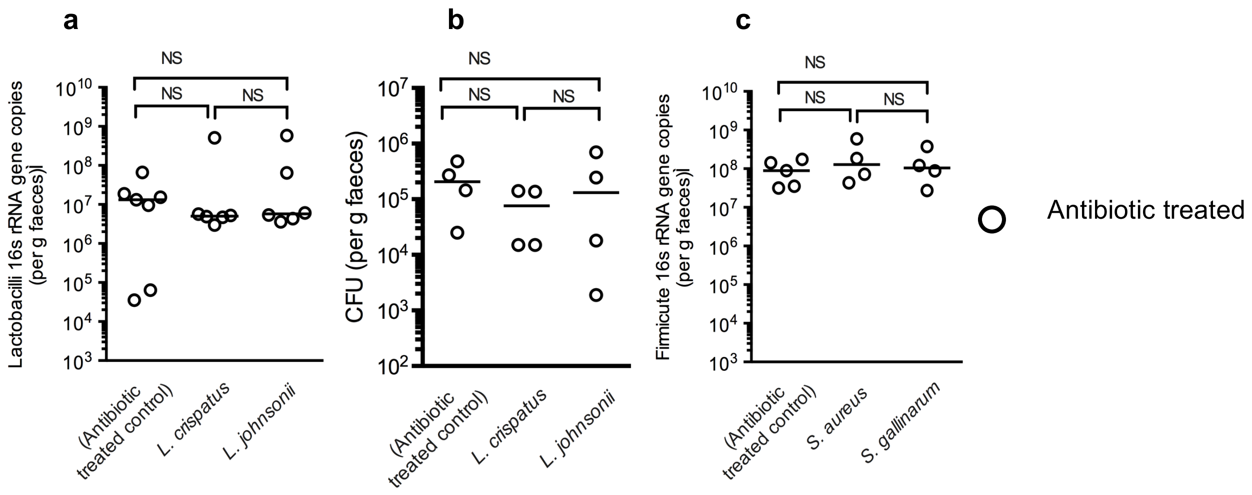

Supplementary Figure 12. Levels of intranasally inoculated airway commensals in the gastrointestinal tract. Lactobacilli levels in faeces of antibiotic treated mice intranasally administered 10^6 CFU of indicated bacteria as measured by 16s qPCR (**a**) or culture on MRS agar (**b**). **c**, Firmicute levels (as a measure of Staphylococcal levels) in faeces of antibiotic treated mice intranasally administered 10^6 CFU of indicated bacteria. Bacterial levels were measured at the termination of lung infection experiment. Each data point represents a single mouse and horizontal lines indicate median value. NS, not significant by one way ANOVA with post hoc Sidak's test.

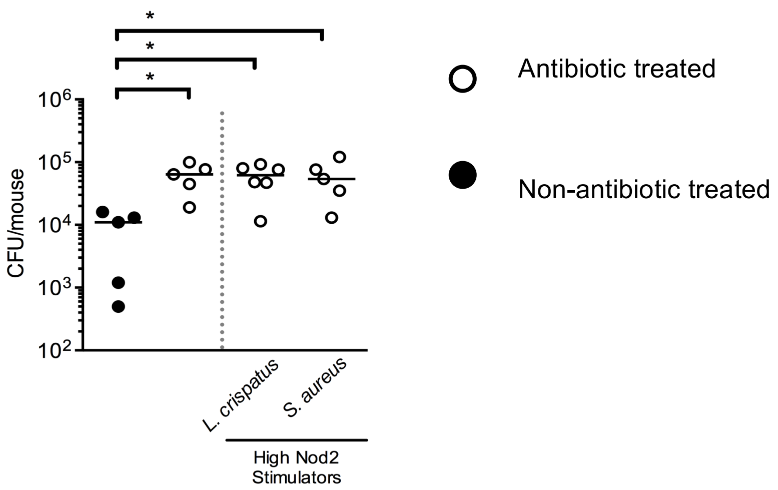

Supplementary Figure 13. Control demonstrating that oral inoculation with 10^6 CFU of the airway commensals into the intestine does no rescue defects in pulmonary bacterial clearance after antibiotic treatment. Indicated groups were orally inoculated with commensals 72 hours prior to *S. pneumoniae* infection. *S. pneumoniae* burden in the lung 12 hours postintranasal inoculation. Each point represents a single mouse and line and horizontal lines indicate median values, *p < 0.05 by one way ANOVA with post hoc Dunnett's test.

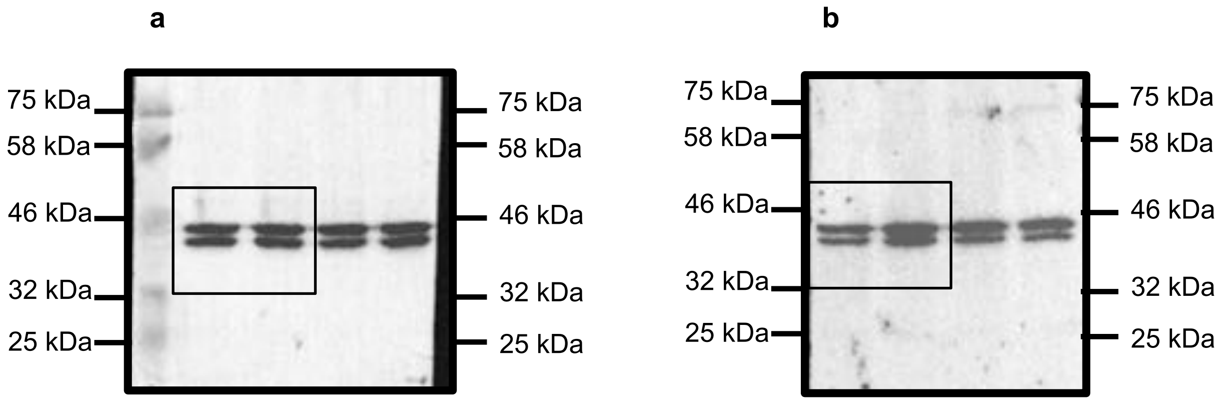

Supplementary Figure 14. Uncropped western blot images used in **Supplementary Fig. 6**, blotting for ERK (a) and blotting for phospho-ERK (b). Sections of blots used in **Supplementary Fig. 6** are highlighted.

Supplementary Table 1

Growth conditions and media for bacteria used in this study

Bacterium	Phylum	Gram-negative or Gram-positive	Growth media	Growth environment
*Bifidobacterium adolescentis**	Actinobacteria	Positive	Reinforced Clostridial broth	Anaerobic, 37°C
Eggerthella lenta	Actinobacteria	Positive	Chopped meat media	Anaerobic, 37°C
*Bifidobacterium breve**	Actinobacteria	Positive	Tryptic soy broth + 5% (v/v) defibrinated Sheep's blood	Anaerobic, 37°C
Bacteroides ovatus	Bacteroidetes	Negative	Supplemented brain heart infusion	Anaerobic, 37°C
Bacteroides fragilis	Bacteroidetes	Negative	Brain heart infusion	Anaerobic, 37°C
Bacteroides thetaiotaomicron	Bacteroidetes	Negative	Tryptic soy broth + 5% (v/v) defibrinated Sheep's blood	Anaerobic, 37°C
Bacteroides uniformis	Bacteroidetes	Negative	Supplemented brain heart infusion	Anaerobic, 37°C
Bacteroides caccae	Bacteroidetes	Negative	Supplemented brain heart infusion	Anaerobic, 37°C
*Bacteroides dorei**	Bacteroidetes	Negative	Clostridial differential broth	Anaerobic, 37°C
Clostridium ramosum	Firmicutes	Positive	Brain heart infusion	Anaerobic, 37°C
*Clostridium symbiosum**	Firmicutes	Positive	Tryptic soy broth + 5% (v/v) defibrinated Sheep's blood	Anaerobic, 37°C
*Clostridium orbiscindens**	Firmicutes	Positive	Chopped meat media	Anaerobic, 37°C
*Lactobacillus reuteri**	Firmicutes	Positive	DeMan, Rogosa and Sharpe broth	Aerobic, 37°C
*Lactobacillus johnsonii**	Firmicutes	Positive	DeMan, Rogosa and Sharpe broth	Aerobic, 37°C
*Lactobacillus rhamnosus**	Firmicutes	Positive	DeMan, Rogosa and Sharpe broth	Aerobic, 37°C
*Lactobacillus crispatus**	Firmicutes	Positive	DeMan, Rogosa and Sharpe broth	Aerobic, 37°C
Enterococcus faecalis	Firmicutes	Positive	Brain heart infusion	Aerobic, 37°C

Eubacterium limosum	Firmicutes	Positive	Chopped meat media	Anaerobic, 37°C
Staphylococcus gallinarum	Firmicutes	Positive	Tryptic soy broth	Aerobic, 37°C
Staphylococcus aureus	Firmicutes	Positive	Tryptic soy broth	Aerobic, 37°C
*Staphylococcus epidermidis**	Firmicutes	Positive	Brain heart infusion	Aerobic, 37°C
Escherichia coli	Proteobacteria	Negative	Luria-Bertani (Lennox) broth	Aerobic, 37°C
Citrobacter koseri	Proteobacteria	Negative	Brain heart infusion	Anaerobic, 37°C
Klebsiella pneumoniae (ATCC 43816)	Proteobacteria	Negative	Luria-Bertani (Lennox) broth	Aerobic, 37°C
Streptococcus pneumoniae (D39)	Firmicutes	Positive	Tryptic soy broth	Aerobic, 37°C

The following reagents were obtained through BEI Resources, NIAID, NIH as part of the Human Microbiome Project: all bacteria marked by *

Supplementary Table 2

Primers used in this study

Primer name	Sequence (5' to 3')	Annealing Temp (^{o}C)	Species used as a template to generate standard curve
16s F qRT	ACTCCTACGGGAGGCAGCAGT	61	*Clostridium symbiosum*
16s R qRT	ATTACCGCGGCTGCTGGC	49.2	
Firmicutes F	GGAGYATGTGGTTTAATTCGAAGCA	63.9	*Clostridium symbiosum*
Firmicutes R	AGCTGACGACAACCATGCAC	66.5	
Bacteroidetes F	GGARCATGTGGTTTAATTCGATGAT	60.4	*Bacteroides uniformis*
Bacteroidetes R	AGCTGACGACAACCATGCAG	66.5	
Lactobacilli F	AGCAGTAGGGAATCTTCCA	58.8	*Lactobacillus reuteri*
Lactobacilli R	AGCAGTAGGGAATCTTCCA	58.7	
mF4/80 F	CTTTGGCTATGGGCTTCCAGTC		
mF4/80 R	GCAAGGAGGACAGAGTTTATCGTG		
mGapdh F	TGTGTCCGTCGTGGATCTGA		
mGapdh R	CCTGCTTCACCACCTTCTTGAT		
